# About One in Five Novice Vapers Buying Their First E-Cigarette in a Vape Shop Are Smoking Abstinent after Six Months

**DOI:** 10.3390/ijerph15091886

**Published:** 2018-08-31

**Authors:** Karolien Adriaens, Dinska Van Gucht, Frank Baeyens

**Affiliations:** 1 Faculty of Psychology and Educational Sciences, KU Leuven–University Leuven, Tiensestraat 102, 3000 Leuven, Belgium; dinska.vangucht@thomasmore.be (D.V.G.); frank.baeyens@kuleuven.be (F.B.); 2 Applied Psychology Unit, Thomas More University of Applied Sciences, Molenstraat 8, 2018 Antwerp, Belgium

**Keywords:** electronic cigarette, vape shop, tobacco harm reduction

## Abstract

*Background*: E-cigarette use is rising with the majority of vapers purchasing their e-cigarettes in vape shops. We investigated the smoking/vaping trajectories and quit-smoking success rates of smokers deciding to start vaping for the first time and buying their e-cigarette in brick-and-mortar vape shops in Flanders. *Methods*: Participants filled out questionnaires assessing smoking/vaping behaviour at three moments (intake, after three and six months) and smoking status was biochemically verified using eCO measurements. *Results*: Participants (*n* = 71) were regular smokers (*M*_eCO-intake_ = 22 ppm), half of whom reported a motivation to quit smoking in the near future. Participants bought 3rd/4th generation e-cigarettes and e-liquid with a nicotine concentration averaging 7 mg/mL. A smoking reduction of 53% (17 cigarettes per day (CPD) at intake to 8 CPD after six months) was observed, whereas eCO decreased to 15 ppm. Eighteen percent of participants had quit smoking completely (eCO = 2 ppm), another 25% had at least halved CPD, whereas 57% had failed to reduce CPD by at least 50% (including 13% lost to follow-up). Quitters consumed more e-liquid than reducers and those who continued to smoke. *Conclusions*: Around one in five smoking customers buying their first e-cigarette in a brick-and-mortar vape shop had quit smoking completely after six months.

## 1. Introduction

Electronic cigarette (e-cigarette) use by former or current smokers is rising across the world, with uptake by never-smokers remaining low [1,2,3]. The most recent numbers show that prevalence varies across countries, with 2% of the 15 years and older EU population currently using e-cigarettes in 2017 [3]. The highest prevalence rates were found in the UK, with almost 6% current vapers [1,4]. In Belgium, around 4% of the population was found to currently use e-cigarettes [3,5]. The most frequently cited reasons by e-cigarette users for taking up and using e-cigarettes are to reduce smoking and/or to quit smoking completely [1,3].

In contrast to traditional smoking cessation tools, such as Nicotine Replacement Therapy (NRT) and smoking cessation medications (e.g., Champix^®^), e-cigarettes are not sold in pharmacies nor prescribed by general practitioners [6]. E-cigarettes are sold in supermarkets, local newsagents and specialized stores, so-called vape shops [4]. Vape shops often offer advice regarding the products they sell and offer generally more sophisticated products (e.g., types of e-cigarettes, e-liquids, accessories) than traditional retail stores [6,7,8]. Therefore, smokers willing to experiment, try out and/or start using e-cigarettes are often advised to buy e-cigarettes in vape shops [6]. The majority of vapers purchase their e-cigarettes in (online) vape shops, rather than for example in local supermarkets [4,6]. In Belgium, we see a manifest rise in brick-and-mortar vape shops [9]. This increase is most likely a result of a recent Royal Decree from 2017, legalizing sales of nicotine-containing e-cigarettes in brick-and-mortar shops while at the same time banning any online sales of hardware and e-liquid [10]. In a recent study done in the UK by Ward and colleagues, results showed that vape shops offer help at the level of the individual customer (e.g., product choice), at the interpersonal level (e.g., lounging area, sociability factor) and at the level of regulations [8]. These examples are specific to brick-and-mortar vape shops and are not present when people purchase e-cigarettes online.

A pivotal question to determine the public health effects of the adoption of vaping by increasing numbers of smokers, is if and to what extent vaping promotes smoking cessation among those who choose to start vaping. Prospective observational cohort studies generally show substantially higher odds of (self-reported) quitting smoking in those who self-select to use e-cigarettes in a quit attempt (OR 2.69–7.88; 20–42% quitters) compared to those who do not [11,12,13,14]. A recurrent observation in those longitudinal cohort studies is that regular (daily) and/or long-term use of efficient e-cigarettes is an important contributor to success. At the same time, both national (UK/France) and EU cross-sectional population data confirm that about half (41–52%) of current daily e-cigarette users report to have quit smoking completely [1,15,16,17]. Due to the very nature of these studies–questionnaire based population surveys–they present no biological verification of self-reported smoking cessation. Moreover, several of these studies only inform us of cumulative and long-term quit-smoking success rates in those smokers who, after the initial choice to start vaping, managed to become regular e-cigarette users. They do not directly speak, however, to the question of the initial smoking/vaping trajectories and quit-smoking success rates of those who decide to start vaping for the very first time.

The majority of smokers who start vaping do so without any kind of professional assistance and many (or most, in countries where online sales are banned) purchase their first e-cigarette in a dedicated (brick-and-mortar) vape shop [4]. The questions of smoking/vaping trajectories and quit-smoking failures or success in vaping novices may be answered by a longitudinal assessment of smokers’ vaping and smoking behaviour after buying their first e-cigarette in a brick-and-mortar vape shop. Therefore, this study focused on first-time e-cigarette use in smoking customers of such shops in Flanders (Dutch speaking part of Belgium). To our knowledge, studies of brick-and-mortar vape shop customers concerning quit smoking rates are scarce, with only one longitudinal study having investigated first-time customers/vapers [18] and two other studies being cross-sectional assessments of vape shop customers [19,20].

Tackett and colleagues assessed vape shop customers’ preferences, harm beliefs, e-cigarette behaviours, smoking history using a questionnaire and biochemically verified smoking status with exhaled carbon monoxide (eCO) measurements [19]. Almost all (90%) approached customers entering the shop participated (*n* = 215) in the study. Most participants (*n* = 208) were found to be (ex-) smokers (97%), experienced users of newer-generation e-cigarettes (78%) and vapers of non-tobacco flavoured e-liquid (59%) with a nicotine concentration of on average 18 mg/mL [18]. Around 66% of those who completed an eCO measurement (*n* = 123) proved to be exclusive e-cigarette users (biochemically verified, eCO ≤ 10 ppm). Dual users (34%) significantly reduced their cigarettes per day (CPD) by 66%. The main reported reason to initiate vaping was to quit smoking (86%) [19].

Wagener and colleagues focused on smoking and vaping behaviours and preferences of 100 vape shop customers [20]. Participation rate was 76% of all entering customers. These customers filled out a questionnaire in the vape shop and performed an eCO measurement to biochemically verify their smoking status. Almost all participants (97%) had been smokers before they had started vaping and were experienced vapers having used e-cigarettes for around 15 months. Of those who performed an eCO measurement (*n* = 91), 62% proved to be exclusive e-cigarette users (biochemically verified, eCO ≤ 10 ppm) [20]. The remaining participants were using both e-cigarettes and tobacco cigarettes (dual use) and had significantly reduced their cigarettes per day (CPD) by 74%. Participants used around eight ml of e-liquid per day, non-tobacco flavoured e-liquid (80%), tank-style e-cigarettes and most (71%) of them had bought their first e-cigarette in a brick-and-mortar vape shop [20]. The most important reported reasons for vaping were to quit or reduce smoking (48%) and to improve health (32%).

Polosa and colleagues were the only researchers so far to conduct a one-year longitudinal study, assessing customers of a brick-and-mortar vape shop buying their first e-cigarette [18]. Results after one year (self-report) showed that 41% of the participants had completely quit smoking, an additional 25% had substantially reduced their number of CPD and 34% had failed to substantially reduce smoking [18].

The studies described above come with several shortcomings. Both Tackett and colleagues and Wagener and colleagues only assessed smoking status at one particular point in time and did not focus on first time customers/e-cigarette users [19,20]. This probably resulted in an over-representation of regular/long-term vapers (those who vape infrequently or quickly give up after starting to vape will show up less in a vape shop than dedicated regular vapers) and no assessment of quit-smoking trajectories of first-time customers was aimed at. Polosa and colleagues did focus on first-time e-cigarette users and included a follow-up period of one year but the study lacked biochemical verification of (quit-) smoking status [18]. In order to address these shortcomings, the current study built on the strengths of previous described studies (e.g., follow-up, biochemical verification) and focused on first-time e-cigarettes users. The main aim was to assess the effect of buying an e-cigarette for the first time on future smoking status. We conducted a six-month longitudinal study assessing smoking and vaping behaviour of first-time e-cigarette users buying their first e-cigarette in a brick-and-mortar vape shop. Participants were questioned at three moments (intake, after three and six months) and smoking status was biochemically verified.

## 2. Materials and Methods

### 2.1. Participants

**Figure 1:**
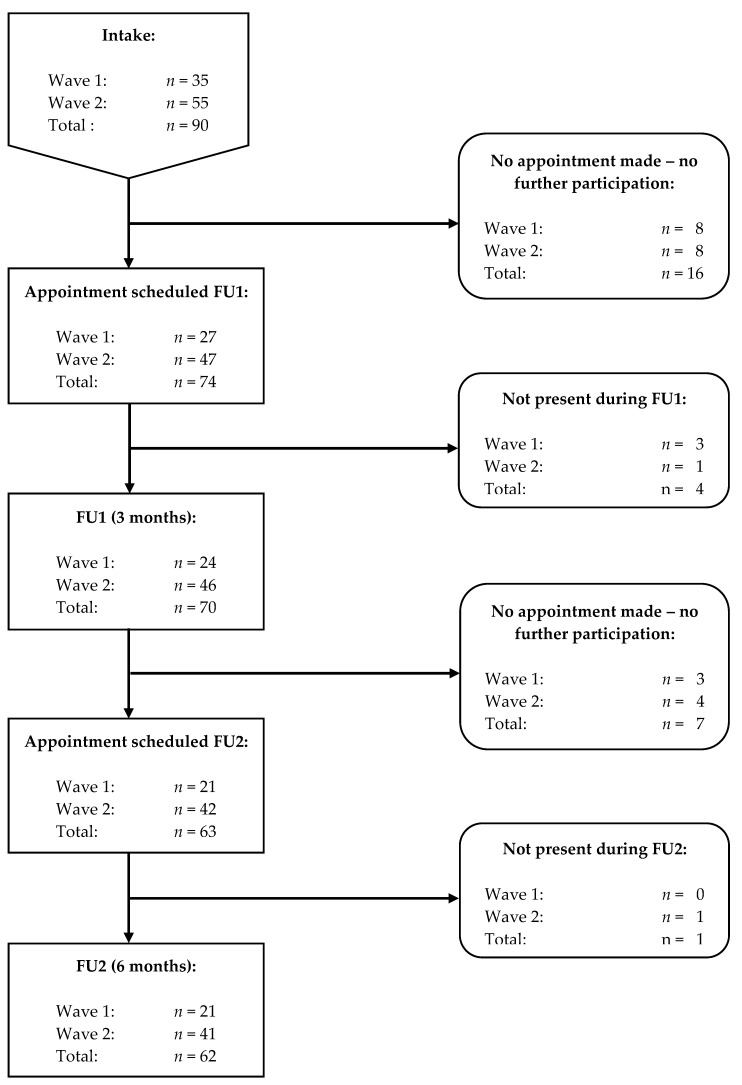
Participant flow; FU = follow-up.

The researcher approached customers in the vape shop during their visit. Customers were eligible when they were older than 18 years, current smokers and buying an e-cigarette for the first time but were excluded when they had ever tried an e-cigarette before. During recruitment, we approached approximately 160 customers of whom eventually 90 were buying their first e-cigarette and agreed to participate in the study (see Figure 1). Throughout the study, 19 participants did not complete any follow-up (FU) measurements, so in total 71 participants with at least one FU measurement were included in all analyses. Sociodemographic characteristics of included participants are shown in Table 1.

### 2.2. Measures

#### 2.2.1. Questionnaires

Two questionnaires were used, one at intake and one at the two FU measurements. The intake questionnaire started with assessing several demographics: age (open ended), gender, highest educational degree, occupation, ethnicity and monthly net income (all predefined categories, with the option to provide additional information; see Table 1). Next, we assessed the “Smoking profile” of participants, including age when having started smoking (open ended), quit smoking attempts (number of attempts and quit-smoking aids used during longest period; predefined categories), current average smoked CPD (open ended), motivation to quit smoking (predefined categories; see Table 2, Results section), harm perception of a tobacco cigarette, e-cigarette, smoking cessation medication and NRT (on Likert scales from 0 “Not harmful at all” to 10 “Very harmful”) and experienced negative health effects of smoking (e.g., “As a smoker I suffer from coughing”; on Likert scales from 0 “Never” to 4 “Always”; see Appendix A, Table A1). The last part questioned the “E-cigarette profile”, including (most important) reasons to purchase an e-cigarette (predefined categories; see Table 3, Results section), future plans regarding e-cigarette use in case of reducing/quitting smoking (predefined categories; see Table 3, Results section), brand and e-cigarette type (open ended), flavour (open ended, see Table 4, Results section) and nicotine concentration of e-liquid (open ended, see Table 4, Results section).

The FU questionnaire contained similar questions regarding the “Smoking profile” and “E-cigarette profile”. For the “Smoking profile”, no additional questions were asked. When participants were still using their e-cigarette (with or without further smoking), they were asked to fill out the “E-cigarette profile” in addition to the “Smoking profile”. The “E-cigarette profile” was extended with the following questions: currently exclusively vaping or both using e-cigarettes and tobacco cigarettes (predefined categories), reasons for preferring an e-cigarette or cigarette (open ended), e-cigarette use (frequency of use and amount of e-liquid consumed per week; open ended), reasons and situations for using an e-cigarette (predefined categories; see Table 3, Results section), experienced negative health effects of vaping (e.g., “As a vaper I suffer from coughing”; on Likert scales from 0 “Never” to 4 “Always”; see Appendix A, Table A2) and benefits of using an e-cigarette (e.g., “Being a vaper helped me to quit smoking”; on Likert scales from 0 “Totally disagree” to 4 “Totally agree”; see Appendix A, Table A3).

#### 2.2.2. Biochemical Verification of Smoking Status

Self-reported smoking status was biochemically verified by measuring eCO levels using a piCO+ Smokerlyzer [21]. Participants with an eCO level above 7 ppm (parts per million) were considered smokers and those with a level of seven or less were considered non-smokers/quitters. eCO levels were measured every time when participants were asked to fill out a questionnaire. In case participants filled out the questionnaire online or by telephone, no eCO levels could be noted (n_FU1_ = 7).

### 2.3. Procedure

To reach a sample of first-time e-cigarette users buying their first e-cigarette in a brick-and-mortar vape shop, we contacted several vape shops in Flanders by telephone to ask if they were willing to cooperate for the recruitment of participants. Five vape shops were prepared to collaborate and received an e-mail with information regarding the study. All shops sold a wide range of e-liquids (varying nicotine concentrations and flavours) and hardware (going from easy to use e-cigarettes to more advanced MODs). The researcher was allowed to sit in the shops for the intake and FU sessions, to recruit participants and collect data.

The recruitment process consisted of two waves, the first one started in December 2015 and ended in July 2016, the second one started in December 2016 and ended in July 2017. The second wave of data collection was added because of the high dropout rate (40%) in the first wave. Each wave consisted of three measurement moments: intake (start), follow-up 1 (FU1; after three months) and follow-up 2 (FU2; after six months).

In sum, the researcher approached potential participants and gave a brief introduction of the six-month longitudinal study. Eligible participants willing to participate signed the informed consent, performed an eCO measurement and filled out the questionnaire. Contact information was collected and arrangements were made for the FU sessions. Those were scheduled after three and six months in the same vape shop. If visiting the vape shop was not feasible for participants, the researcher proposed and arranged to schedule a visit at the house of the participant. Reminders for the sessions were sent by e-mail. In case of no response of the participants, they were contacted by telephone (maximum three times). Participants who completed all three questionnaires received 25 euro as a compensation for their participation. The Societal and Social Ethics Committee of the University of Leuven approved the research protocol (G-2015 11 380).

### 2.4. Statistical Analyses

First, excluded participants (*n* = 19) were compared to included participants (*n* = 71) regarding all variables measured at intake. For these comparisons, we used Pearson’s chi square tests (categorical variables) or Fisher’s exact tests (when the expected n was below five in more than 20% of the cells) and one-way ANOVAs (continuous variables). Second, analyses for most variables measured at intake and FU regarding “Smoking profile” and “E-cigarette profile” included descriptive statistics (means, standard deviations, frequencies). Reduction rates were calculated as well for FU1 and FU2 (see Section 3.4.1). Third, we calculated correlations to check if specific participants’ characteristics at intake could predict smoking status at FU2. Fourth, to compare results on categorical variables between intake, FU1 and FU2, Pearson’s chi square tests or Fisher’s exact tests were used. To check for changes in continuous variables (e.g., eCO levels) from intake to FU1 and FU2, we used repeated measurement ANOVAs, whether or not using a categorical variable (e.g., reduction rates at FU2) as between subject variable and moment as a within-subjects variable. Last, alpha levels of 0.05 were used and all analyses were performed using Statistica, version 13 (TIBCO Software Inc., Palo Alto, CA, USA) [22].

## 3. Results

Excluded participants (*n* = 19) did not differ from included participants (*n* = 71) for all variables measured at intake (all *p*’s > 0.06), except for education level, χ^2^(5) = 12.68, *p* = 0.03 and experienced negative health effects as a smoker, F(1, 88) = 7.03, *p* = 0.01. Those who were included at FU had at least a non-academic university degree (college degree, 34%) and they experienced less negative health effects (M = 1.60, SD = 0.57) compared to excluded participants (22% with a non-academic university degree; M = 2.03, SD = 0.80, respectively).

### 3.1. Smoking Profile at Intake

Participants on average started smoking at the age of 15 (SD = 2.65) and most (82%) had tried to quit smoking for on average three times (SD = 2.89) in the past. The longest quit smoking attempt had lasted on average seven months (SD = 13.80) with the shortest being less than one month and the longest eight years. Most participants had tried to quit smoking on willpower (72%), or with the use of medication (19%) or NRT (19%). Less common smoking cessation aids were counselling (7%) and alternative strategies (7%).

At intake, participants smoked on average 17 CPD (SD = 9.67) and had an eCO level of 22 ppm (SD = 11.08). The three main reported reasons to smoke were out of habit (73%), because of craving for a cigarette (66%) and to reduce stress (61%). Participants mainly smoked after a meal (83%), when drinking (73%) and at home (66%), see Table 2. Negative health effects from smoking were in general seldom to occasionally experienced (M = 1.60, SD = 0.57), with a bad condition (M = 2.54, SD = 0.91), pondering about their health (M = 2.17, SD = 1.25) and cough tendencies (M = 2.03, SD = 1.05) rated highest (see Appendix A, Table A1). Around half of the participants (49%) reported a short-term motivation to quit smoking, meaning they wanted to quit smoking immediately (32%) or wanted to quit smoking in the following month (17%). The other half of participants (51%) displayed a long-term motivation to quit smoking: 32% wanted to quit smoking in the following six months, 15% wanted to quit smoking but not yet in the following six months and 3% did not want to quit smoking, see Table 2.

At intake, harm perception differed significantly for the different products (cigarette, smoking cessation medication, NRT, e-cigarette), F(3, 210) = 119.71, *p* < 0.001. Cigarettes (M = 8.69, SD = 1.29) were perceived as most harmful, followed by smoking cessation medication (M = 5.10, SD = 2.13) and NRT (M = 4.82, SD = 2.36). E-cigarettes (M = 3.54, SD = 1.69) were perceived as least harmful for health.

### 3.2. E-Cigarette Profile at Intake

Participants main motivations to purchase an e-cigarette were to quit smoking by gradually reducing smoking (52%), to quit smoking by immediately switching completely to vaping (27%) and to reduce smoking (11%; dual use), see Table 3. Most people had no additional reasons for their purchase (39%); those who did mainly indicated curiosity (23%), reducing smoking (20%), quitting smoking (13%) and vaping where smoking is prohibited (13%). Thus, for some smokers reducing or quitting smoking was not the main reason to start vaping but rather an additional reason. In case participants would reduce or quit smoking, 45% wanted to continue using the e-cigarette but reduce the nicotine concentration, 25% wanted to quit using the e-cigarette, 24% wanted to reduce using the e-cigarette and 6% wanted to continue using the e-cigarette without reducing the nicotine concentration in the future, see Table 3.

All participants purchased 3rd/4th generation e-cigarettes (i.e., refillable and rechargeable e-cigarettes with adjustable settings such as wattage, temperature, etc.) and mainly chose e-liquid with a sweet (65%), tobacco (46%) or mint (25%) flavour. On average, a nicotine concentration of 7 mg/mL (SD = 2.75) was bought with a minimum of 0 and a maximum of 12 mg/mL.

### 3.3. Predictors Smoking Status at FU

We checked if specific participant characteristics at intake (i.e., eCO level, CPD, age start smoking, number of years smoking, number of quit smoking attempts, longest period quit smoking, motivation to quit smoking, experienced negative health effects from smoking and nicotine concentration at intake) could predict smoking status (i.e., eCO level and CPD) at FU2. Only eCO level and CPD at intake significantly correlated both with eCO level (r = 0.36 and r = 0.51, respectively) and CPD (r = 0.28 and r = 0.46, respectively) at FU2.

### 3.4. Smoking Status and Profile at FU

#### 3.4.1. Smoking Status at FU

**Figure 2:**
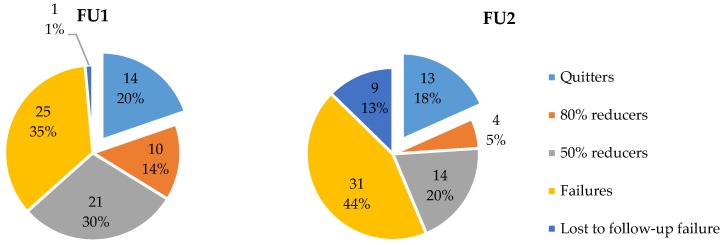
Reduction rates at FU1 and FU2.

Smoking status was measured at FU1 and FU2 by the number of reported smoked CPD. At FU1 20% of participants had completely quit smoking and 79% was still smoking (1% was lost to FU). At FU2, similar results were obtained, 18% quitters and 69% still smoking, respectively; although the number of participants lost to FU was higher (13%). The reduction rates were calculated as well, more specifically: people who completely quit smoking (and eCO ≤ 7 ppm) were considered as “Quitters”; those who reduced their number of CPD with 80% or more (but less than 100%) were considered as “80% reducers”; people who reduced their number of CPD 50% or more (but less than 80%) were considered as “50% reducers”; and those who smoked more than 50% of their baseline CPD were considered as “Failures”. Participants with no data about CPD at FU were considered as “Lost to FU failures”. Figure 2 graphically displays the distribution of the different categories at FU1 and at FU2. The distribution significantly differed between the two FUs, χ^2^(4) = 11,05, *p* = 0.03.

**Figure 3:**
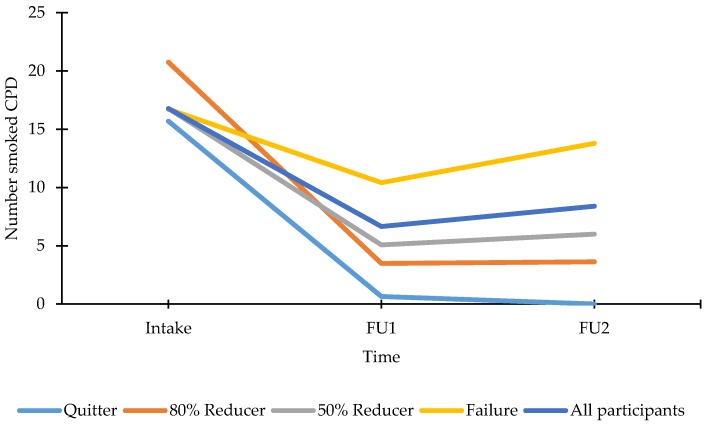
Smoked CPD at intake, FU1, FU2 for all participants and reduction rate groups as calculated at FU2. Note: All participants: n_intake–FU1–FU2_ = 61, M_intake_ (SE between brackets) = 16.78 (1.27), M_FU1_ = 6.65 (0.92), M_FU2_ = 8.39 (0.98); Quitters: n_intake–FU1–FU2_ = 13, M_intake_ = 15.69 (2.81), M_FU1_ = 0.65 (1.70), M_FU2_ = 0.00 (1.46); 80% reducers: n_intake–FU1–FU2_ = 4, M_intake_ = 20.75 (5.07), M_FU1_ = 3.48 (3.06), M_FU2_ = 3.63 (2.62); 50% reducers: n_intake–FU1–FU2_ = 14, M_intake_ = 16.79 (2.71), M_FU1_ = 5.07 (1.64), M_FU2_ = 6.00 (1.40); Failures: n_intake–FU1–FU2_ = 30, M_intake_ = 16.72 (1.85), M_FU1_ = 10.42 (1.12), M_FU2_ = 13.78 (0.96).

The number of CPD for the total group of participants showed a significant decrease, F(1, 60) = 86.61, *p* < 0.001, from 17 CPD at intake, to 7 CPD at FU1 and subsequently an increase to 8 CPD at FU2, F(1, 60) = 10.12, *p* < 0.01 (see Figure 3). Participants who became quitters, 80% reducers, 50% reducers or failures at FU2, did not differ with respect to CPD at intake (all *p*’s > 0.39). All the reduction rate groups at FU2 showed a similar pattern over time, namely a significant decrease in CPD from intake to FU1 (all *p*’s < 0.001) followed by a stagnation to FU2 (all F’s < 1), except Failures showing a significant increase from FU1 to FU2, F(1, 57) = 21.06, *p* < 0.001 (but still showing a significant reduction of CPD from intake to FU2, F(1, 57) = 4.49, *p* < 0.05). The exact numbers of CPD for each group at each moment are presented in Figure 3.

**Figure 4:**
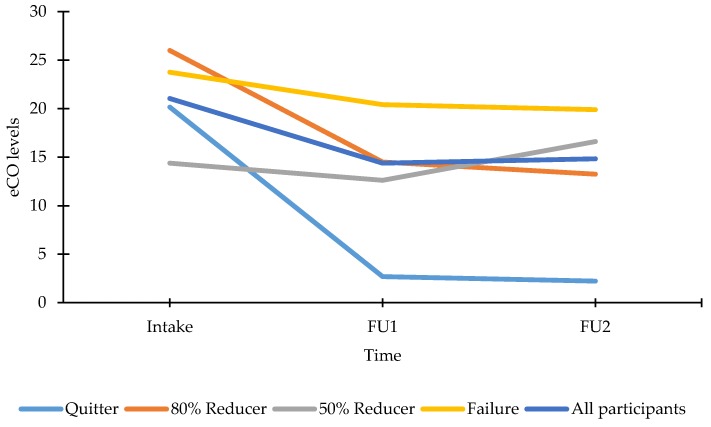
eCO measurements at intake, FU1, FU2 for all participants and reduction rate groups as calculated at FU2. Note: All participants: n_intake–FU1–FU2_ = 59, M_intake_ (SE between brackets) = 21.05 (1.47), M_FU1_ = 14.39 (1.59), M_FU2_ = 14.83 (1.50); Quitters: n_intake–FU1–FU2_ = 13, M_intake_ = 20.15 (3.01), M_FU1_ = 2.69 (2.84), M_FU2_ = 2.23 (2.60); 80% reducers: n_intake–FU1–FU2_ = 4, M_intake_ = 26.00 (5.43), M_FU1_ = 14.50 (5.12), M_FU2_ = 13.25 (4.68); 50% reducers: n_intake–FU1–FU2_ = 13, M_intake_ = 14.38 (3.01), M_FU1_ = 12.62 (2.84), M_FU2_ = 16.62 (2.60); Failures: n_intake–FU1–FU2_ = 29, M_intake_ = 23.76 (2.02), M_FU1_ = 20.41 (1.90), M_FU2_ = 19.90 (1.74).

eCO levels showed similar trends as the reported smoked CPD. The eCO levels for all participants showed a significant decrease, F(1, 58) = 14.66, *p* < 0.001, from 21 ppm at intake to 14 ppm at FU1 and subsequently a stagnation, F < 1, at 15 ppm at FU2 (see Figure 4). At intake, eCO levels did not differ between reduction rate groups defined at FU2 (all *p*’s > 0.07), except for those who continued smoking at FU2 showing a significantly higher eCO level at intake compared to 50% reducers, F(1, 55) = 6.68, *p* < 0.05. From intake to FU1, Quitters were the only ones showing significantly lower eCO levels, F(1, 55) = 26.85, *p* < 0.001. Quitters (M_FU2_ = 2.23 ppm) and 80% reducers (M_FU2_ = 13.25 ppm) were the only groups showing a reduced eCO level from intake to FU2 (both *p*’s < 0.05; 50% reducers and Failures F < 1 and *p* > 0.05, respectively). The exact eCO levels for each group at each moment are presented in Figure 4.

#### 3.4.2. Smoking Profile at FU

In general, those who still smoked at FU1 (*n* = 56) and FU2 (*n* = 49) showed a largely similar smoking profile (e.g., situations when smoking, reasons why smoking, experienced negative health effects from smoking; for the latter see Appendix A, Table A1) compared to the total group at intake, see Table 2. Remarkably, eight in thirteen quitters at FU2 reported a long-term motivation to quit smoking at intake (i.e., willing to quit smoking in the following six months or later), whereas only five quitters wanted to quit smoking in the near future (i.e., following month or sooner).

The harm perception for the different products (cigarette, smoking cessation medication, NRT, e-cigarette) did not change over time, F(6, 354) = 1.44, *p* = 0.20. Across all measurements, cigarettes (M = 8.67, SE = 0.16) were perceived as most harmful, followed by smoking cessation medication (M = 4.64, SE = 0.25) together with NRT (M = 4.42, SE = 0.26). E-cigarettes (M = 3.60, SE = 0.21) were perceived as least harmful for health. The harm perception for e-cigarettes did not change over time, F < 1, or differ between the different reduction rate groups, F(3, 56) = 2.05, *p* = 0.12.

### 3.5. Vaping Status and E-Cigarette Profile at FU

#### 3.5.1. Vaping Status at FU

Vaping status was measured at FU1 and FU2 by questioning participants if they were using an e-cigarette and if so, if they were exclusively vaping or both vaping and smoking (dual use). At FU1 (*n* = 71) 77% was using an e-cigarette, with 20% exclusively vaping and 57% both using cigarettes and e-cigarettes. The remaining 23% was not vaping anymore. The distribution of participants (not) vaping was more or less the same at FU2 (*n* = 62): 68% was vaping, with 21% exclusively vaping and 47% dual using. Reported reasons for quitting e-cigarette use included no satisfaction (FU1: 31%; FU2: 30%), uncomfortable practical use (FU1: 25%; FU2: 35%), not sufficiently similar to smoking cigarettes (FU1: 25%; FU2: 15%), physical complaints from e-cigarette use (FU1: 13%; FU2: 15%), no confidence in efficacy of e-cigarettes (FU1: 13%; FU2: 0%) and no reason given (FU1: 6%; FU2: 15%), see Table 3.

**Figure 5:**
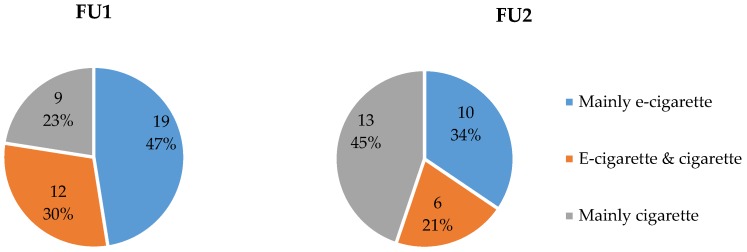
Distribution dual use at FU1 and FU2. Note: Proportion of cigarette/e-cigarette use was classified as “Mainly cigarette (e-cigarette)” as soon as reported proportion of cigarette (e-cigarette) use exceeded 60%; other responses were categorized as “E-cigarette & cigarette”.

Figure 5 shows the usage patterns of dual users at FU1 and FU2: around half of the dual users were mainly (more than 60% of the time) using an e-cigarette at FU1, whereas at FU2 half of the dual users were mainly using cigarettes. The main reported reasons for preferring a cigarette over an e-cigarette was the aspect of addiction and that smoking was a habit, see Table 3. The preference for using an e-cigarette centred around health aspects (including for others’ health) and the taste/smell.

#### 3.5.2. E-Cigarette Profile at FU

In general, participants were using 3rd/4th generation e-cigarettes. All user characteristics are shown in Table 4. Quitters at FU2 consumed (across both FUs) significantly more e-liquid per week compared to reducers and those who continued to smoke (all *p*’s < 0.01). No effects were observed regarding frequency of e-cigarette use (reported puffs per day), F(3, 32) = 1.38, *p* = 0.27. At the end of the study vapers used slightly lower nicotine concentrations than at intake (7 mg/mL at intake to 6 mg/mL at FU2), F(2, 76) = 4.58, *p* < 0.05, with no difference between reduction rate groups from FU2, F(3, 35) = 1.18, *p* = 0.33. The e-cigarette profile of those still vaping at FU1 (*n* = 55) and FU2 (*n* = 42) was similar for both moments (e.g., situations when vaping, reasons why vaping, future plans regarding vaping in case reducing or quit smoking), see Table 3. Almost no negative health effects from vaping (*n* = 41) were reported at both FU measurements (M_FU1_ = 0.66, M_FU2_ = 0.69) and this remained stable over time, F < 1 (see Appendix A, Table A2). Experienced benefits from vaping were rated higher compared to experienced negative health effects (M_FU1_ = 2.69, M_FU2_ = 2.64), with no change over time, F < 1 (see Appendix A, Table A3).

## 4. Discussion

The main aim of this study was to conduct a longitudinal and detailed assessment of smokers’ vaping and smoking behaviour after buying their first e-cigarette in a brick-and-mortar vape shop. A pivotal question to answer in the context of Tobacco Harm Reduction is if and to what extent vaping promotes smoking cessation among those who choose to start vaping. Even though both well-conducted observational cohort studies and (inter)national cross-sectional population surveys leave no doubt that smokers who self-select to use e-cigarettes and vape regularly (and/or long-term) show substantially higher odds of self-reported quitting (or substantially reduced smoking), these studies are also characterized by several shortcomings and lacunae that are inherent to the very nature of their research designs [1,11,12,13,14,15,16,17,18,19,20]. Importantly, apart from not including a biological verification of self-reported smoking cessation, they generally do not allow a fine-grained assessment of transitioning smokers’ experiences, perceptions and attitudes. To our knowledge, the current study is one of the first to longitudinally assess these behaviours in an elaborate manner, starting from the very first experiences with vaping and including biochemical verification of self-reported smoking cessation.

The main results obtained in this sample of regular smokers (*n* = 71; *M*_CPD-intake_ = 17; *M*_eCO-intake_ = 22 ppm) recruited in five vape shops in Flanders, included a general reduction of 53% in CPD across all participants from intake to the end of the study (after six months), going from 17 CPD to 8 CPD. Overall, eCO levels decreased as well from 21 ppm at intake to 15 ppm at the end of the study. In the current study, 18% of participants had completely quit smoking after six months, another 25% had at least halved baseline CPD, whereas 57% had failed to reduce CPD by at least 50% (including 13% lost to follow-up). Unlike smoking, vaping resulted in almost no reported negative health effects. No clear predictors (e.g., motivation to quit smoking or nicotine concentration at intake) of pronounced smoking reduction or successful smoking cessation were found; however, eCO levels and CPD at intake showed some correlation with quit-smoking rates at the end of the study. In comparison with our results, Polosa and colleagues found higher quit-smoking rates after six months: 42% completely quit smoking and 30% substantially reduced smoking and 28% failed to substantially reduce smoking [18]. A possible explanation for this difference in quit-smoking rates could be the lack of biochemical verification of participants’ smoking status in the study of Polosa, allowing for the possibility that not all self-reported quitters may have been completely smoke-free [18]. It seems unlikely, however, that the difference in quit rates in both studies should be attributed more than in part to such eventual “cheating” behaviour. Namely, a recent estimate of the accurateness of self-reported smoking abstinence in e-cigarette users (based on urinary NNAL, a biomarker of exposure to tobacco-specific nitrosamine NNK) came to the conclusion that no more than about 1 in 6 vapers reporting only using e-cigarettes may also smoke (unreported) tobacco cigarettes [23]. It is unclear, however, to what extent other factors (such as possible differences between both samples in quit-smoking motivation, level of smoking/nicotine dependence, type of e-cigarette used, information provided about adequate use of e-cigarette) may have contributed to the lower quit rates obtained in the current study. Yet, participants in the Polosa study tended to use higher nicotine concentrations, tobacco flavoured liquid and initially smoked more CPD [18].

Cross-sectional studies assessing vape shop customers also typically found higher biochemically verified quit-smoking rates, ranging from 62 to 66% [19,20]. This probably reflects that most customers of vape shops are long-term e-cigarette users who have quit smoking (those who vape infrequently or quickly give up after starting to vape will show up less in a vape shop than dedicated regular vapers), which could explain the higher rates obtained in these cross-sectional studies [7].

However, putting the quit-smoking rates obtained in the current study into perspective, the results are still higher compared to quit-smoking rates when smokers quit smoking only relying on willpower, NRT or even smoking cessation medication [15,24]. Long-term (6 to 12 months) quit-smoking rates when using no smoking cessations tools are typically around 3–5% [25], whereas the rates for NRT and smoking cessation medication no more than double or at best triple the success compared to “willpower” alone [26,27]. In addition, results obtained in quit-smoking services in England showed that only 8% of clients of smoking services maintained smoking abstinence after one year [28].

These results indicate, that for those smokers who voluntary choose to try out vaping (with or without intention to quit smoking), benefits can be expected to occur regarding smoking behaviour. For some smokers, complete smoking abstinence was not achieved but they were able to reduce the amount of smoked CPD by becoming “dual users” (combined smoking and vaping). In our study, 57% of participants were dual users at FU1 and 47% at FU2. These results seem to be in line with those of population data, where currently it is found that around 50% of vapers are dual users [1,29]. Although dual users reduced smoking with 47% (see Figure 3), reduced smoking is not without any risks. It is important for vapers to be aware that reducing the amount of smoked CPD to smoking only a few cigarettes a day still has a negative impact on health and that for many smoking-related diseases the risk reduction is not a linear function of the reduction of the amount smoked [30,31,32]. A recent study showed that long-term use of only e-cigarettes resulted in significantly reduced levels of smoking related carcinogens and toxins, whereas this was not observed in those who were both using e-cigarettes and tobacco cigarettes; highlighting the importance of completely switching to vaping [33]. On the other hand, dual use is, for some smokers, part of the trajectory to become a complete switcher [34] and probably the biggest benefit of reduced smoking (in this case, by dual using) may be that it increases the likelihood of achieving future cessation [35]. In this respect, there is a possible role for vape shop owners to help and guide smoking customers to completely quit smoking by switching to vaping exclusively. Because salespeople of a vape shop are often the first to whom smokers will be asking for advice [4,6,7], it is important that they are well trained to give the best customer-driven advice and information [8]. Research does show that salespeople are already taking up such a consultative role when it comes to guiding and advising customers regarding several (technical) aspects of vaping and smoking cessation [36]. However, it is also important to keep in mind that, for some smokers, e-cigarettes may not be satisfying enough to ever be acceptable as an exclusive smoking substitute [37].

The main motivations of participants to purchase an e-cigarette were to reduce or quit smoking. In line with this, participants bought 3rd/4th generation e-cigarettes but noteworthy, e-liquid with a nicotine concentration of on average no more than 7 mg/mL. This nicotine concentration is in the same range as observed in two other studies in Flemish/Dutch vapers, where online vape shop customers (eight in 10 complete switchers) used on average 9.7 mg/mL of nicotine and 4–8 mg/mL of nicotine was the most frequent choice in both dual users and switchers of another more recent convenience sample [38,39]. However, when taking into account not only the nicotine concentration but also the volume of e-liquid consumed per week, it must be noted that the overall volume of about 13 mL/week in the current study is substantially lower than the 19.5 mL/week in the Van Gucht and colleagues sample and definitely lower than the 38.1 mL/week in the Adriaens and colleagues sample (23.7 mL/week in dual users, 41.4 mL/week in switchers) [38,39]. It can therefore be questioned if the total nicotine intake per week (by means of vaping) was sufficiently high for vaping plausibly becoming a satisfactory alternative source of nicotine delivery for the majority of participants of the current study. This view is reinforced by the observation that successful switchers consumed almost double the amount of e-liquid (21–22 mL/week) than the total group average.

Vapers (including dual users) at FU1 and FU2 reported a willingness to reduce the nicotine concentration still further when having reduced or quit smoking. This is remarkable because only about one in five participants had already succeeded in complete smoking cessation, whereas a majority were dual users still “needing” a couple of cigarettes a day. A possible explanation for this phenomenon could be the fear of nicotine. E-cigarettes by themselves were perceived as much less harmful compared to tobacco cigarettes, possibly implying that the wish to reduce the nicotine concentration originates from a fear of nicotine rather than from a fear of vaping (absent nicotine). The (little) existing literature shows that this trend of using low nicotine concentrations and wanting to reduce the concentrations is rising [38,40]. Besides the fear of nicotine, some vapers decrease their nicotine levels because of financial reasons, health reasons, or willingness to quit vaping in the long term [41]. Therefore, it is important to highlight that, over time, even long-term vapers can show changes in their vaping behaviours [41]. Again, there is an important role for salespeople of vape shops to be well educated regarding nicotine concentrations, to take away the potential fear of nicotine and to give the best possible advice to smoking customers.

The results obtained in this longitudinal assessment of first-time e-cigarette users should be seen in the light of the following limitations. First, we used a convenience sample of smoking customers buying their first e-cigarette in one of a limited number of selected brick-and-mortar vape shops. Consequently, one should be cautious to generalize to the experiences of smokers/novice-vapers buying a first e-cigarette at other “physical” vape shops, or starting their vaping trajectory as online vape shop customer. Second, it would represent a category mistake to interpret our findings as the equivalent of a randomized controlled trial on using e-cigarettes for smoking cessation: it seems highly likely that smokers who decide to try vaping are self-selecting for having a better-than-average chance it will work for them [42]. Third, although we recruited participants in two waves, the sample size remains relatively low and the follow-up period was limited to six months. Fourth, the interpretation of the results should be seen in light of the exclusion criteria used. For the analyses, we excluded those participants who did not fill out at least one follow-up. If those would be included, attrition rates at FU2 would increase to 31% instead of 13% and quit rates at FU2 would decrease to 14% instead of 18%. Fifth and last, the current study was not designed to determine predictors of successful switching to vaping.

## 5. Conclusions

Overall, the results of the current study among smokers buying their first e-cigarette in a brick-and-mortar vape shop indicate that around one in five was able to quit smoking completely by switching to vaping and an additional one in four to reduce the number of CPD to at least half of the habitual CPD. Smokers who bought e-cigarettes in a brick-and-mortar vape shop in Flanders were using 3rd/4th generation e-cigarettes, although the nicotine concentrations used and the amount of e-liquid consumed were relatively low. The resulting low nicotine consumption (obtained from vaping) could possibly and partially explain the relatively low quit-smoking rates. To the extent that this interpretation is valid, salespeople of vape shops might wish to advice novice vapers to use e-liquid with sufficiently high nicotine concentrations, and/or not to be afraid of vaping frequently and as much as needed or desired.

## Figures and Tables

**Table 1:** Sociodemographic characteristics of included participants.

Variable	*n*	M (SD) or %
**Demographic characteristics**	**71**	
Age (years)	71	38.25 (14.06)
Gender (men/women)	44/27	61.97/38.03
**Highest educational degree**	**70**	
None	2	2.86
Elementary school	4	5.71
High school	40	57.14
Non-academic bachelor	16	22.86
University	5	7.14
Other	3	4.29
**Occupation**	**71**	
Student	7	9.86
Part-time job	2	2.82
Full-time job	52	73.24
Housewife/-man	1	1.41
Job seeker	1	1.41
Retired	6	8.45
Invalidity	2	2.82
**Net income per month (in €)**	**70**	
<1000 €	10	14.29
1000–1500 €	13	18.57
1500–2000 €	30	42.86
2000–2500 €	13	18.57
2500–3000 €	3	4.29
>3000 €	1	1.43
**Ethnicity**	**71**	
Caucasian	70	98.59
Other	1	1.41

**Table 2:** Smoking profile: situations when smoking, reasons why smoking, motivation to quit smoking and experienced negative health effects from smoking at intake, follow-up 1 (FU1) and follow-up 2 (FU2).

Variable	*n*_intake (%)_	*n*_FU1 (%)_	*n*_FU2 (%)_
Situations when smoking *	**71**	**56**	**49**
After a meal	59 (83.10)	34 (60.71)	31 (63.27)
When drinking (coffee, alcohol)	52 (73.24)	30 (53.57)	29 (59.19)
At home	47 (66.20)	26 (46.43)	24 (48.98)
With friends	45 (63.38)	19 (33.93)	17 (34.69)
At work or school	38 (53.52)	20 (35.71)	19 (38.78)
Alone	37 (52.11)	18 (32.14)	16 (32.65)
Reasons why smoking *	**71**	**56**	**49**
Routine	52 (73.24)	24 (42.86)	22 (44.90)
Craving	47 (66.20)	37 (66.07)	28 (57.14)
Stress reduction	43 (60.56)	26 (46.43)	26 (53.06)
Atmosphere	33 (46.48)	17 (30.36)	20 (40.82)
Nicotine craving	29 (40.85)	15 (26.79)	12 (24.49)
Relaxation	29 (40.85)	14 (25.00)	14 (28.57)
Pastime	20 (28.17)	9 (16.07)	11 (22.45)
**Motivation to quit smoking**	**71**	**56**	**48**
I do not want to quit smoking	2 (2.82)	5 (8.93)	5 (10.42)
I think about quitting smoking but not in the next 6 months	11 (15.49)	12 (21.43)	14 (29.17)
I think about quitting smoking, in the next 6 months	23 (32.39)	21 (37.50)	17 (35.42)
I think about quitting smoking, in the next month	12 (16.90)	6 (10.71)	8 (16.67)
I want to quit smoking now	23 (32.39)	12 (21.43)	4 (8.33)
Experienced negative health effects from smoking **	**46**	**46**	**46**
	1.63 (0.09)	1.52 (0.08)	1.46 (0.08)

Note: * multiple responses were possible; ** *M* (*SE*).

**Table 3:** E-cigarette profile: most important reason e-cigarette purchase, future plans e-cigarette in case reduced/quit smoking, reasons quit vaping, dual use preference e-cigarette, dual use preference cigarette, situations when vaping and reasons why vaping.

Variable	*n*_intake (%)_	*n*_FU1 (%)_	*n*_FU2 (%)_
**Most important reasons e-cigarette purchase**	**71**	**/**	**/**
To quit smoking–gradually reducing smoking	37 (52.11)	/	/
To quit smoking–immediately switching	19 (26.76)	/	/
To reduce smoking (dual use)	8 (11.27)	/	/
Curiosity	3 (4.23)	/	/
To vape where smoking is prohibited	1 (1.41)	/	/
Financial reasons	0 (0.00)	/	/
No reason indicated	3 (4.23)	/	/
**Future plans e-cigarette in case reduced/quit smoking**	**71**	**54**	**43**
Continue using e-cigarette, without reducing nicotine concentration	4 (5.63)	8 (14.81)	10 (23.26)
Continue using e-cigarette and reducing nicotine concentration	32(45.07)	32 (39.51)	16 (37.21)
Reducing e-cigarette use	17 (23.94)	7 (12.96)	10 (23.26)
Quitting e-cigarette use	18 (25.35)	7 (12.96)	7 (16.28)
Reasons why quit vaping *	**/**	**16**	**20**
No satisfaction	/	5 (31.25)	6 (30.00)
Uncomfortable practical use	/	4 (25.00)	7 (35.00)
Little similarities with cigarettes	/	4 (25.00)	3 (15.00)
Physical complaints from e-cigarette use	/	2 (12.50)	3 (15.00)
No confidence in e-cigarettes	/	2 (12.50)	0 (0.00)
No reason reported	/	1 (6.25)	3 (15.00)
Dual use–preference e-cigarette *	**/**	**41**	**29**
Health	/	18 (43.90)	13 (44.83)
Context	/	9 (21.95)	6 (20.69)
For others	/	5 (12.20)	2 (6.90)
Financial aspect	/	2 (4.88)	0 (0.00)
Taste/smell	/	11 (26.83)	3 (10.34)
Smoking cessation	/	1 (2.44)	5 (17.24)
Other or no reason	/	6 (14.63)	2 (6.90)
Dual use–preference cigarette *	**/**	**41**	**29**
Sensorial aspects of smoking cigarettes	/	6 (14.63)	9 (31.03)
Context	/	11 (26.83)	5 (17.24)
Stress	/	5 (12.20)	2 (6.90)
Addiction	/	10 (24.39)	8 (27.59)
Habit	/	9 (21.95)	9 (31.03)
Technical problems e-cigarette	/	2 (4.88)	1 (3.45)
Other or no reason	/	4 (9.76)	0 (0.00)
Situations when vaping *	**/**	**55**	**42**
At home	/	35 (63.64)	26 (61.90)
At work/school	/	26 (47.27)	21 (50.00)
With friends	/	20 (36.36)	18 (42.86)
After a meal	/	18 (32.73)	7 (16.67)
Everywhere	/	15 (27.27)	9 (21.43)
When drinking (coffee, alcohol)	/	14 (25.46)	11 (26.19)
Alone	/	14 (25.46)	12 (28.57)
Reasons why vaping *	**/**	**55**	**42**
Craving for e-cigarette	/	29 (52.73)	19 (45.24)
Nicotine craving	/	28 (50.91)	18 (42.86)
Routine	/	22 (40.00)	11 (26.19)
Stress reduction	/	19 (34.55)	10 (23.81)
Relaxation	/	12 (21.82)	12 (28.57)
Atmosphere	/	11 (20.00)	11 (26.19)
Pastime	/	9 (16.36)	8 (19.05)

Note: * multiple responses were possible.

**Table 4:** User characteristics vapers: puffs per day, amount of e-liquid and nicotine concentration used, flavour e-liquid.

Variable	*n* ^a^	Intake	FU1	FU2
**Puffs per day**	**36**	**/**	**77.67 (10.75)**	**83.30 (27.20)**
Quitters	11	/	126.93 (17.20)	142.05 (47.67)
80% reducers	4	/	30.38 (28.52)	66.63 (79.05)
50% reducers	8	/	60.06 (20.17)	165.13 (55.89)
Failures	13	/	61.38 (15.82)	24.37 (43.85)
**Amount of e-liquid used (mL/week)**	**37**	**/**	**12.95 (1.60)**	**13.14 (1.68)**
Quitters	11	/	21.36 (2.50)	22.45 (2.51)
80% reducers	4	/	6.50 (4.14)	13.75 (4.16)
50% reducers	9	/	9.50 (2.76)	9.89 (2.77)
Failures	13	/	10.19 (2.30)	7.21 (2.31)
**Nicotine concentration (mg/mL)**	**39**	**7.01 (0.41)**	**6.01 (0.51)**	**5.79 (0.56)**
Quitters	12	7.00 (0.75)	5.63 (0.95)	4.50 (0.98)
80% reducers	3	6.00 (1.49)	5.00 (1.89)	3.50 (1.96)
50% reducers	10	6.35 (0.82)	5.60 (1.04)	5.90 (1.07)
Failures	14	7.71 (0.69)	6.86 (0.88)	7.29 (0.91)
**Flavour e-liquid**		**71 ^a^**	**55 ^a^**	**41 ^a^**
Tobacco ^b^		33 (46.48)	15 (27.27)	14 (34.15)
Mint ^b^		18 (25.35)	15 (27.27)	9 (21.95)
Coffee ^b^		6 (8.45)	3 (5.55)	2 (4.88)
Sweet ^b^		46 (64.79)	40 (72.73)	28 (68.29)

Note: all M (SE) except for ^a,b^, ^a^ are *n*, ^b^ are *n* (%).

## Appendix A

**Table A1:** Experienced negative health effects from smoking at intake, FU1 and FU2.

Variable	Moment	Never *n (*%)	Seldom *n (*%)	Occasionally *n* (%)	Often *n (*%)	Always *n (*%)	*n* M (SD)
Increased weight	IN	44 (62.86)	10 (14.29)	11 (15.71)	4 (5.71)	1 (1.43)	70 0.69 (1.03)
	FU1	31 (56.57)	12 (35.71)	8 (25.00)	2 (3.64)	2 (3.64)	55 0.76 (1.07)
	FU2	23 (46.94)	14 (28.57)	8 (16.33)	0 (0.00)	4 (8.16)	49 0.94 (1.18)
Headaches	IN	32 (45.07)	20 (28.17)	12 (16.90)	7 (9.86)	0 (0.00)	71 0.92 (1.01)
	FU1	16 (28.57)	26 (46.43)	11 (19.64)	3 (5.36)	0 (0.00)	56 1.02 (0.84)
	FU2	17 (34.69)	14 (28.57)	17 (34.69)	1 (2.04)	0 (0.00)	49 1.04 (0.89)
Sleeping problems	IN	37 (52.11)	13 (18.31)	10 (14.08)	7 (9.86)	4 (5.63)	71 0.99 (1.26)
	FU1	23 (41.07)	19 (33.93)	5 (8.93)	8 (14.29)	1 (1.79)	56 1.02 (1.12)
	FU2	22 (44.90)	16 (32.65)	8 (16.33)	3 (6.122)	0 (0.00)	49 0.84 (0.92)
Increased palpitations	IN	20 (28.17)	21 (29.58)	19 (26.76)	8 (11.27)	3 (4.23)	71 1.34 (1.13)
	FU1	16 (28.57)	20 (35.71)	14 (25.00)	5 (8.93)	1 (1.79)	56 1.20 (1.02)
	FU2	12 (24.49)	22 (44.90)	12 (24.49)	3 (6.12)	0 (0.00)	49 1.12 (0.86)
Poor sense of smell	IN	20 (28.57)	15 (21.43)	21 (30.00)	13 (18.57)	1 (1.43)	70 1.43 (1.34)
	FU1	14 (25.00)	20 (35.71)	9 (16.07)	12 (21.43)	1 (1.79)	56 1.39 (1.14)
	FU2	12 (24.49)	20 (40.82)	13 (26.53)	3 (6.12)	1 (2.04)	49 1.20 (0.96)
Poor sense of taste	IN	12 (17.39)	19 (27.54)	26 (37.68)	12 (17.39)	0 (0.00)	69 1.55 (0.98)
	FU1	10 (17.86)	23 (41.07)	16 (28.57)	6 (10.71)	1 (1.79)	56 1.38 (0.96)
	FU2	7 (14.29)	17 (34.69)	22 (44.90)	2 (4.08)	1 (2.04)	49 1.45 (0.87)
Poor taste sensation	IN	16 (22.86)	14 (20.00)	25 (35.71)	13 (18.57)	2 (2.86)	70 1.59 (1.22)
	FU1	11 (19.64)	21 (37.50)	16 (28.57)	6 (10.71)	2 (3.57)	56 1.41 (1.04)
	FU2	6 (12.25)	22 (44.90)	18 (36.73)	2 (4.08)	1 (2.04)	49 1.39 (0.84)
Unpleasant smelling	IN	12 (17.14)	21 (30.00)	16 (22.86)	17 (24.29)	4 (5.71)	70 1.71 (1.18)
	FU1	4 (7.28)	24 (43.64)	12 (21.82)	12 (21.82)	3 (5.45)	55 1.75 (1.06)
	FU2	6 (12.50)	21 (43.75)	13 (27.08)	5 (10.42)	3 (6.25)	48 1.54 (1.05)
Dry throat	IN	12 (16.90)	18 (25.35)	23 (32.39)	14 (19.72)	4 (5.63)	71 1.71 (1.14)
	FU1	7 (12.50)	20 (35.71)	21 (37.50)	7 (12.50)	1 (1.79)	56 1.55 (0.93)
	FU2	6 (12.25)	17 (34.69)	19 (38.78)	7 (14.29)	0 (0.00)	49 1.55 (0.89)
Dry mouth	IN	8 (11.27)	21 (29.58)	24 (33.80)	13 (18.31)	5 (7.04)	71 1.80 (1.09)
	FU1	7 (12.50)	20 (35.71)	17 (30.36)	11 (19.64)	1 (1.79)	56 1.63 (1.00)
	FU2	7 (14.29)	15 (30.61)	21 (42.86)	6 (12.25)	0 (0.00)	49 1.53 (0.89)
Difficult breathing	IN	8 (11.43)	14 (20.00)	31 (44.29)	16 (22.86)	1 (1.43)	70 1.83 (0.96)
	FU1	6 (10.71)	15 (26.79)	24 (42.86)	9 (16.07)	2 (3.57)	56 1.75 (0.98)
	FU2	6 (12.25)	11 (22.45)	23 (46.94)	8 (16.33)	1 (2.04)	49 1.73 (0.95)
Cough tendencies	IN	5 (7.14)	17 (24.29)	24 (34.29)	19 (27.14)	5 (7.14)	70 2.03 (1.05)
	FU1	4 (7.27)	14 (25.45)	21 (38.18)	15 (27.27)	1 (1.82)	55 1.91 (0.95)
	FU2	4 (8.16)	16 (32.65)	16 (32.65)	12 (24.49)	1 (2.04)	49 1.80 (0.98)
Pondering health	IN	10 (14.08)	10 (14.08)	19 (26.76)	22 (30.99)	10 (14.08)	71 2.17 (1.25)
	FU1	6 (10.71)	12 (21.43)	18 (32.14)	15 (26.79)	5 (8.93)	56 2.02 (1.14)
	FU2	5 (10.20)	9 (18.37)	20 (40.82)	13 (26.53)	2 (4.08)	49 1.96 (1.02)
Bad condition	IN	1 (1.41)	9 (12.68)	20 (28.17)	33 (46.48)	8 (11.27)	71 2.54 (0.91)
	FU1	2 (3.64)	8 (14.55)	18 (32.73)	20 (36.36)	7 (12.73)	55 2.40 (1.01)
	FU2	2 (4.08)	6 (12.25)	18 (36.73)	16 (32.65)	7 (14.29)	49 2.41 (1.02)

Note: all *n* (%) unless otherwise specified; IN = intake; items ordered based on Means intake from low to high.

**Table A2:** Experienced negative health effects from vaping at FU1 and FU2.

Variable	Moment	Never *n (*%)	Seldom *n (*%)	Occasionally *n (*%)	Often *n (*%)	Always *n (*%)	*n* M (SD)
Headaches	FU1	39 (70.91)	14 (25.45)	2 (3.64)	0 (0.00)	0 (0.00)	55 0.33 (0.55)
	FU2	27 (64.29)	9 (21.43)	6 (14.29)	0 (0.00)	0 (0.00)	42 0.50 (0.74)
Poor sense of smell	FU1	37 (67.27)	17 (30.91)	1 (1.82)	0 (0.00)	0 (0.00)	55 0.35 (0.52)
	FU2	26 (61.90)	16 (38.10)	0 (0.00)	0 (0.00)	0 (0.00)	42 0.38 (0.49)
Unpleasant smelling	FU1	35 (63.64)	17 (30.91)	3 (5.46)	0 (0.00)	0 (0.00)	55 0.42 (0.60)
	FU2	28 (66.67)	13 (30.95)	1 (2.38)	0 (0.00)	0 (0.00)	42 0.36 (0.53)
Sleeping problems	FU1	36 (65.46)	15 (27.27)	3 (5.46)	1 (1.82)	0 (0.00)	55 0.44 (0.69)
	FU2	27 (64.29)	12 (28.57)	2 (4.76)	1 (2.38)	0 (0.00)	42 0.45 (0.71)
Increased palpitations	FU1	35 (63.64)	14 (25.46)	4 (7.27)	2 (3.64)	0 (0.00)	55 0.51 (0.79)
	FU2	25 (59.52)	12 (28.57)	4 (9.52)	1 (2.38)	0 (0.00)	42 0.55 (0.77)
Poor sense of taste	FU1	32 (58.18)	17 (30.91)	5 (9.09)	0 (0.00)	1 (1.82)	55 0.56 (0.81)
	FU2	23 (54.76)	16 (38.10)	2 (4.76)	1 (2.38)	0 (0.00)	42 0.55 (0.71)
Difficult breathing	FU1	27 (50.00)	22 (40.74)	4 (7.40)	1 (1.85)	0 (0.00)	54 0.61 (0.71)
	FU2	23 (54.76)	13 (30.95)	3 (7.14)	3 (7.14)	0 (0.00)	42 0.67 (0.90)
Bad condition	FU1	27 (49.09)	23 (41.82)	3 (5.45)	2 (3.63)	0 (0.00)	55 0.64 (0.75)
	FU2	20 (47.62)	17 (40.48)	3 (7.14)	0 (0.00)	2 (4.76)	42 0.74 (0.96)
Poor taste sensation	FU1	27 (49.09)	18 (32.73)	9 (16.36)	1 (1.82)	0 (0.00)	55 0.71 (0.81)
	FU2	15 (35.71)	22 (52.38)	3 (7.14)	2 (4.76)	0 (0.00)	42 0.81 (0.77)
Increased weight	FU1	30 (54.55)	16 (29.09)	5 (9.09)	3 (5.46)	1 (1.82)	55 0.71 (0.98)
	FU2	23 (56.10)	9 (21.95)	4 (9.76)	3 (7.32)	2 (4.88)	41 0.83 (1.18)
Dry throat	FU1	27 (50.00)	14 (25.93)	10 (18.52)	3 (5.56)	0 (0.00)	54 0.80 (0.94)
	FU2	15 (35.71)	15 (35.71)	8 (19.05)	2 (4.76)	2 (4.76)	42 1.07 (1.09)
Dry mouth	FU1	28 (50.91)	13 (23.64)	10 (18.18)	4 (7.27)	0 (0.00)	55 0.82 (0.98)
	FU2	19 (45.24)	12 (28.57)	7 (16.67)	2 (4.76)	2 (4.76)	42 0.95 (1.13)
Pondering health	FU1	26 (47.27)	16 (29.09)	9 (16.36)	3 (5.46)	1 (1.82)	55 0.85 (1.01)
	FU2	17 (40.48)	12 (28.57)	9 (21.43)	3 (7.41)	1 (2.38)	42 1.02 (1.07)
Cough tendencies	FU1	16 (29.09)	17 (30.91)	14 (25.46)	6 (10.91)	2 (3.64)	55 1.29 (1.12)
	FU2	15 (35.71)	14 (33.33)	8 (19.05)	3 (7.14)	2 (4.76)	42 1.12 (1.13)

Note: all *n* (%) unless otherwise specified; items ordered based on Means intake from low to high.

**Table A3:** Experienced benefits from vaping at FU1 and FU2.

Variable	Moment	Totally Disagree *n (*%)	Disagree *n (*%)	Neutral *n (*%)	Agree *n (*%)	Totally Agree *n (*%)	*n* M (SD)
Not disturbing others	FU1	4 (7.55)	10 (18.87)	15 (28.01)	11 (20.75)	13 (24.53)	53 2.36 (1.26)
	FU2	0 (0.00)	12 (28.57)	7 (16.67)	13 (30.95)	10 (23.81)	42 2.50 (1.15)
No technical problems	FU1	2 (3.64)	12 (21.82)	7 (12.73)	19 (34.55)	15 (27.27)	55 2.60 (1.21)
	FU2	0 (0.00)	9 (21.95)	8 (19.51)	14 (34.15)	10 (24.39)	41 2.61 (1.09)
Helps to quit smoking	FU1	1 (1.82)	5 (9.09)	12 (21.82)	18 (32.73)	19 (34.55)	55 2.89 (1.05)
	FU2	1 (2.50)	2 (50.00)	10 (25.00)	13 (32.50)	14 (35.00)	40 2.93 (1.03)
Helps to take away craving cigarette	FU1	2 (3.64)	3 (5.46)	9 (16.36)	19 (34.55)	22 (40.00)	55 3.02 (1.06)
	FU2	1 (2.44)	4 (9.76)	11 (26.83)	9 (21.95)	16 (39.02)	41 2.85 (1.13)
More places	FU1	5 (9.09)	14 (25.46)	13 (23.64)	10 (18.18)	13 (23.64)	55 3.22 (1.32)
	FU2	5 (11.90)	8 (19.05)	7 (16.67)	14 (33.33)	8 (19.05)	42 2.29 (1.31)

Note: all *n* (%) unless otherwise specified; items ordered based on Means intake from low to high.
